# Supplementary material for: DNA damage-induced PARP1 activation confers cardiomyocyte dysfunction through NAD+ depletion in experimental atrial fibrillation
Source: Nat Commun. 2019 Mar 21;10:1307. doi: 10.1038/s41467-019-09014-2 (PMC6428932; doi:10.1038/s41467-019-09014-2)

DNA damage-induced PARP1 activation confers cardiomyocyte dysfunction through NAD^+ depletion in Atrial Fibrillation

Zhang et al.

Supplementary Information

Supplementary Methods

Optical voltage mapping in HL-1 cardiomyocytes

Action potential (AP) generation was investigated on monolayer cultures of HL-1 cardiomyocytes by optical voltage mapping using 16 µM di-4-ANEPPS (Thermo Fisher Scientific) as fluorescent voltage indicator. Tachypacing induced paucity in fluorescent signals in dispersed areas across the cell culture, indicating excessive reduction or even absence of action potential generation, precluding proper assessment of depolarization velocities in the cell monolayer [1]. In short, HL-1 cardiomyocytes were seeded in 24-well cell culture plates on bovine fibronectin-coated round glass coverslips (diameter of 15 mm). Confluent monolayers of HL-1 cardiomyocytes were incubated for 12 h in medium containing 20 µM olaparib, 40 µM ABT-888 or vehicle DMSO, followed by non-pacing or tachypacing (5 Hz, 40 V, 20 ms) for 8 h in the continued presence of treatment. Optical signals were captured using a MiCAM ULTIMA-L imaging system (SciMedia) and analyzed using Brain Vision Analyzer 1208 software (Brainvision). Noise artefacts were minimized by averaging of the signals at a selected pixel and its eight nearest neighbors. AP duration (APD) at 30% and 80% repolarization (APD_{30} and APD_{80}, respectively), and APD dispersions at different percentages of repolarization were only determined in HL-1 cultures showing full capture after 1-Hz electrical stimulation via a STG 2004 stimulus generator and MC Stimulus II software (both from Multi Channel Systems). Quantitative analyses of excited area of each monolayer culture were performed with the Java-based image processing program Image J (version 1.50i, National Institutes of Health).

Algorithm of Drosan software

To process the heart tube signal *x*, low pass filter is utilized. The used low pass moving average filter of 2N+1 point is:

$$y_i = \frac{1}{2N+1}\sum_{k=-N}^{N} x_{i+k}$$

Default setting is *N=5*. The detection of the start of a beat is the maximum detection in the derivative of the filtered signal *y*, calculated as:

$$dy_i = \frac{1}{2}(y_{i+1} - y_{i-1})$$

Next, find a dy_i with dy_i>*TriggerLevel* and then find the first *i* with $dy_i > dy_{i+1}$. Then, *i* is the sample at the start of the beat. Start the detection algorithm again after *i+nInh* samples. nInh is the number of samples corresponding with the inhibition period (default setting is 200 ms).

The *TriggerLevel* is set to 1.25 SD_y by default, and can be manually adjusted in the user interface. After detection of the beats, valid signal segments are selected (maximal 3 segments). The outcome parameters, as listed in Supplementary Table 2, are calculated for each segment and for the combined segments.

Heart wall movies were also analyzed by using SOHA software developed by Cammarato A *et a l* [2].

Supplementary Tables

Supplementary Table 1. Overview of protective effects of PARP inhibitors and NAD^+ replenishment in tachypaced HL-1 cardiomyocytes, rat atrial cardiomyocytes and *Drosophila melanogaster*

**Drugs**	**targets**	**IC50**	**Conc. in HL-1**	**Conc. in adult rat atrial CM**	**Conc. in *Drosophila***	**Protection in CM**	**Protection in *Drosophila***
3-AB	PARPs		3 mM	n.a.	30 mM	+	-
ABT-888	PARP1 PARP2	5.2 nM PARP1, 2.9 nM PARP2[3]	5-40 µM	5 µM	200, 400 µM	+	+
Olaparib	PARP1 PARP2	5 nM PARP1, 1 nM PARP2[4]	20 µM	5 µM	400 µM	+	+
NAD^+	-		0.25-1 mM	n.a.	5,10 mM	+	+

-, no significant protective effect; +, significant protective effect (*P*<0.05 or *P*<0.01 vs control TP), n.a., data not available.

SupplementaryTable 2. Overview of the outcome parameters calculated with the Drosan software

Variable	Description	Unit
**Nbts**	Number of beats	-
**mIBI**	Mean interbeat interval	ms
**sdIBI**	SDNN or standard deviation of interbeat interval	ms
**minIBI**	Shortest interbeat interval	ms
**maxIBI**	Longest interbeat interval	ms
**rMSSD**	Root mean square of sucessive differences of interbeat interval	ms
**HR**	Heart rate (=*60000/mIBI*)	beats per min
**medianIBI**	Median of IBI values	ms

Note that heart period (HP) is denoted here as Interbeat Interval (IBI).

Supplementary Table 3. Baseline demographic and clinical characteristics of patients with AF and control patients in SR

	**SR**	**AF**
N	11	14
RAA (*n*)	11	14
LAA (*n*)	5	5
Age (mean, std)	72 ± 8	71 ± 4
Months since diagnosis (median, range)	–	14.6 (8.3-36)
Persistent AF (*n*)	–	6
Longstanding persistent AF (*n*)	–	8
**Underlying heart disease (*n*) / surgical procedure**		
Mitral valve surgery	6 (55%)	10 (71%)
Aortic Valve surgery	2 (14%)	4 (29%)
CABG	4 (36%)	1 (7%)
**Medication (*n*)**		
ACE/ARB	6 (55%)	11 (79%)
Digoxin	0 (0%)	4 (29%)
Ca^{2+} channel blocker	0 (0%)	1 (7%)
β-blocker	6 (55%)	9 (64%)

SR: sinus rhythm, AF: atrial fibrillation, RAA: right atrial appendages, LAA: left atrial appendages, CABG: coronary artery bypass grafting, ACE: angiotensin-converting enzyme, ARB: angiotensin receptor blocker, β-Blocker: beta-adrenergic antagonists.

References

1. Askar, S. F. *et al*. Antiproliferative treatment of myofibroblasts prevents arrhythmias in vitro by limiting myofibroblast-induced depolarization. *Cardiovasc. Res.* **90**, 295-304 (2011).

2. Cammarato, A., Ocorr, S. & Ocorr, K. Enhanced assessment of contractile dynamics in Drosophila hearts. *BioTechniques* **58**, 77-80 (2015).

3. Donawho, C. K. et al. ABT-888, an orally active poly(ADP-ribose) polymerase inhibitor that potentiates DNA-damaging agents in preclinical tumor models. Clin. Cancer Res. 13, 2728-2737 (2007)

4. Menear, K. A. *et al*. 4-[3-(4-cyclopropanecarbonylpiperazine-1-carbonyl)-4-fluorobenzyl]-2H-phthalazin- 1-one: a novel bioavailable inhibitor of poly(ADP-ribose) polymerase-1. *J. Med. Chem.* **51**, 6581-6591 (2008).

Supplementary Figures

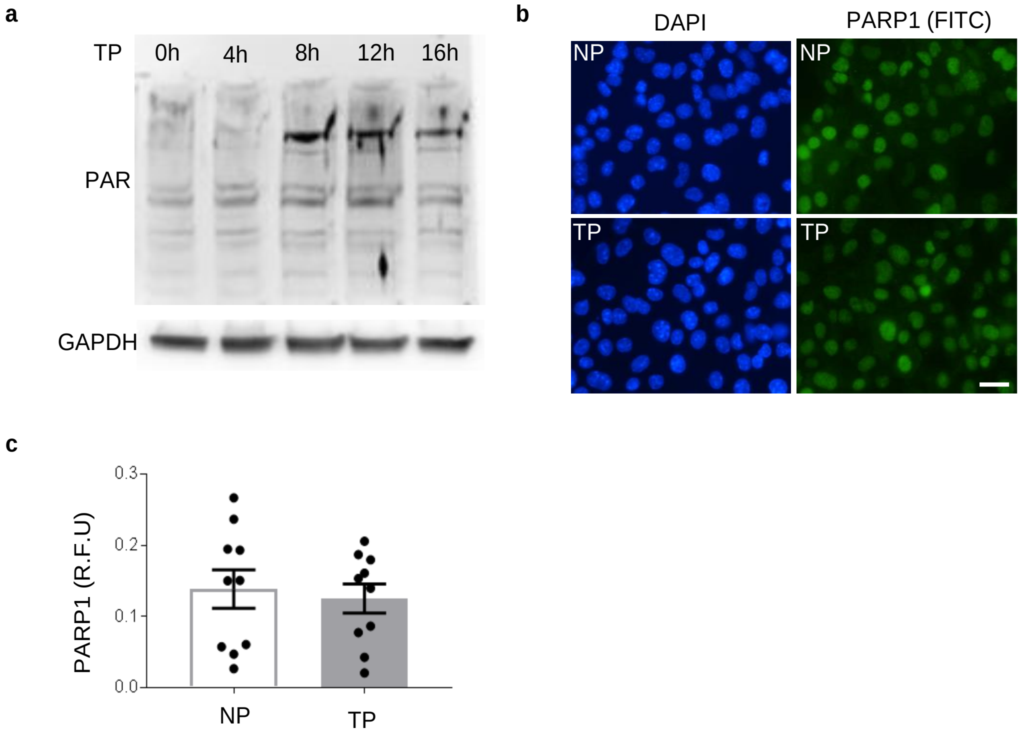

Supplementary Figure 1: Tachypacing, but not PARP1 overexpression, induces PARP activation in HL-1 cardiomyocytes. **a**) Representative original Western blot showing gradual PAR induction especially at 220 KDa during tachypacing (TP) for the time periods as indicated. **b**) and **c**) Immunofluorescence staining and quantified data of PARP1 in control (non-paced, 0 h), and in 12 h TP HL-1 cardiomyocytes. No significant difference was found in the amount of PARP1. N=10 images from over 300 cardiomyocytes. Scalebar is 15 µm. Data are presented as mean ± s.e.m. and two-tailed t-test was used to evaluate differences between groups.

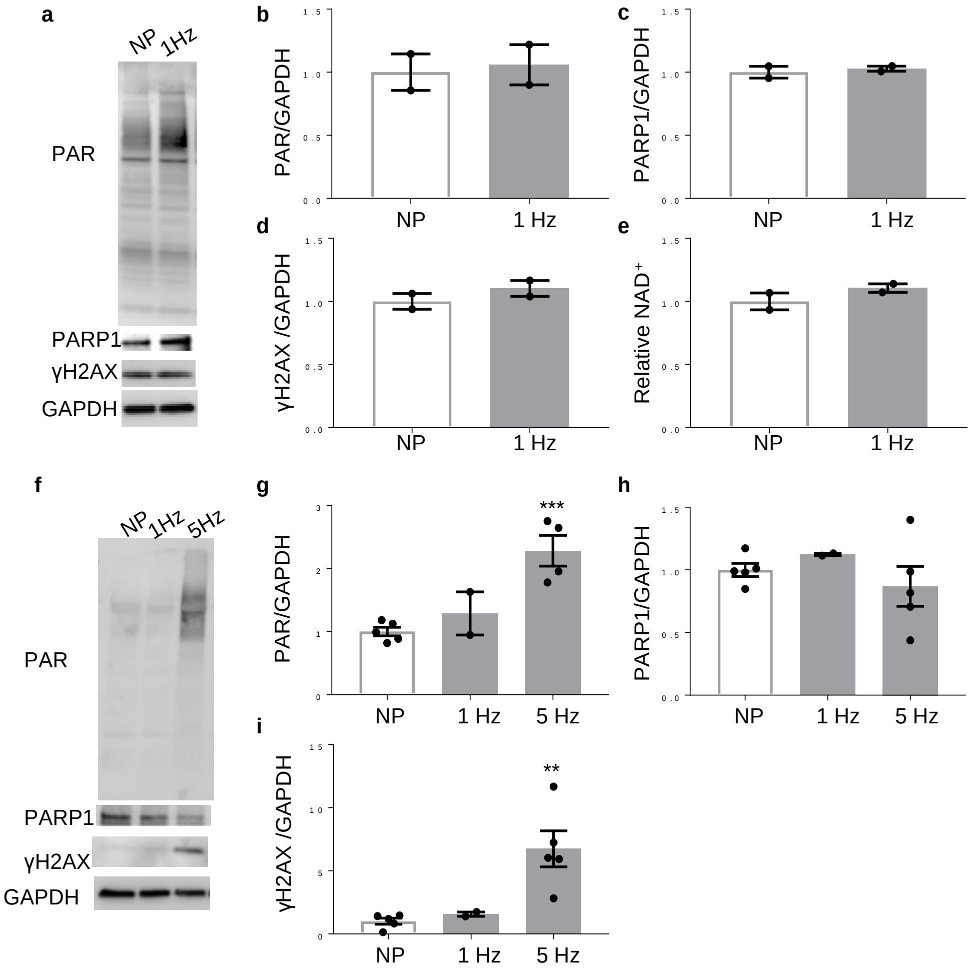

Supplementary Figure 2: Normal pacing (1 Hz) did not induce PARP1 activation nor NAD$^+$ depletion. a-d) Representative Western blot and quantified data showing similar PAR and γH2AX levels in 1 Hz normal paced compared to control non-paced (NP) HL-1 cardiomyocytes. **e**) No difference of NAD$^+$ levels between NP and 1 Hz paced HL-1 cardiomyocyte. **f-i**) Representative Western blot and quantified data showing comparable PAR and γH2AX levels in 1 Hz normal paced rat atrial cardiomyocytes compared to control non-paced (NP) rat atrial cardiomyocytes while tachypacing (5 Hz) significantly induced PAR and γH2AX levels indicating activation of PARP1 and DNA damage, respectively. $^{**}P<0.01$, $^{***}P<0.001$ vs NP. N=5 independent experiments for NP and 5Hz, N=2 independent experiments for 1 Hz. Data are presented as mean ± s.e.m. and two-tailed t-test was used to evaluate differences between groups.

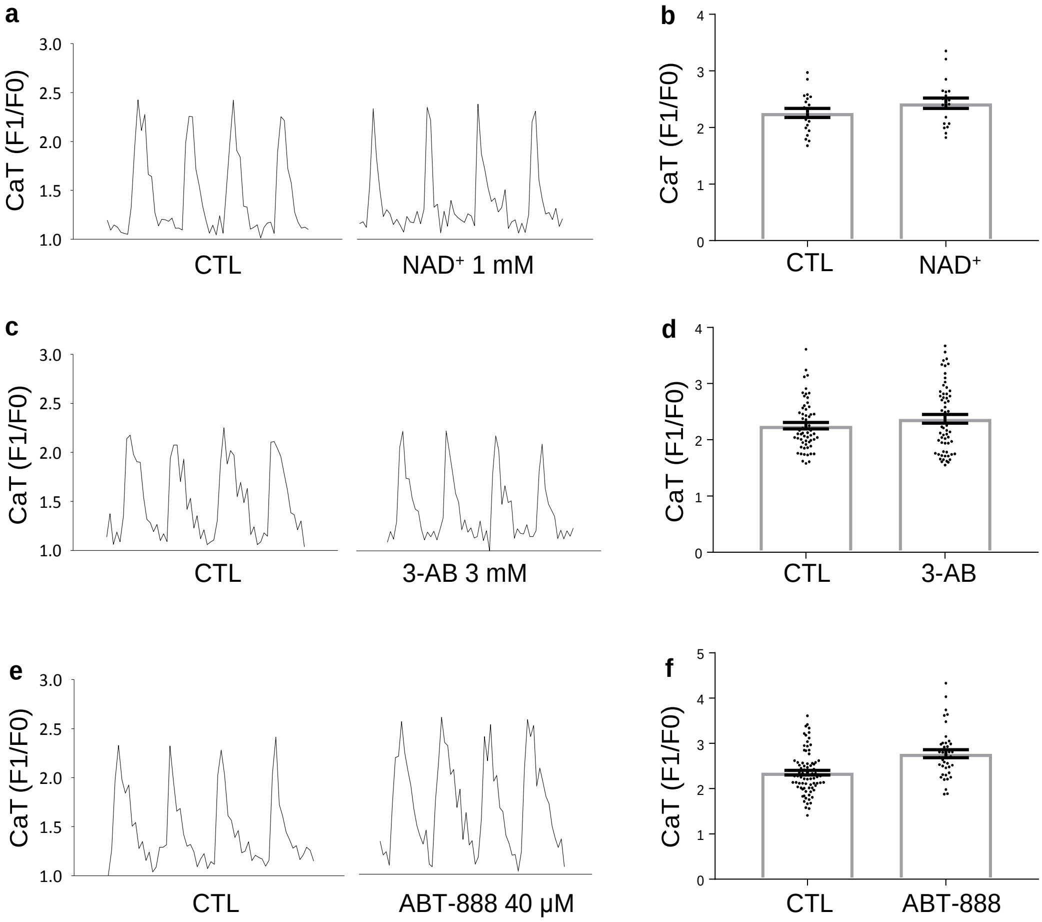

Supplementary Figure 3: NAD$^+$ and PARP1 inhibitors do not influence baseline CaT in HL-1 cardiomyocytes. **a**) and **b**) Representative CaT traces and quantified CaT data of normal paced HL-1 cardiomyocytes treated with NAD$^+$ (1 mM) or vehicle (Control). No changes in CaT were observed between the groups. **c**) and **d**) Representative CaT traces and quantified CaT data in normal paced HL-1 cardiomyocytes treated with 3 AB (3 mM) or vehicle (Control). No changes were observed between the groups. **e**) and **f**) Representative CaT traces and quantified CaT data demonstrate no significant changes in normal paced HL-1 cardiomyocytes treated with ABT-888 (40 μM) compared to vehicle (CTL). Data are presented as mean ± s.e.m. and two-tailed t-test was used to evaluate differences between groups.

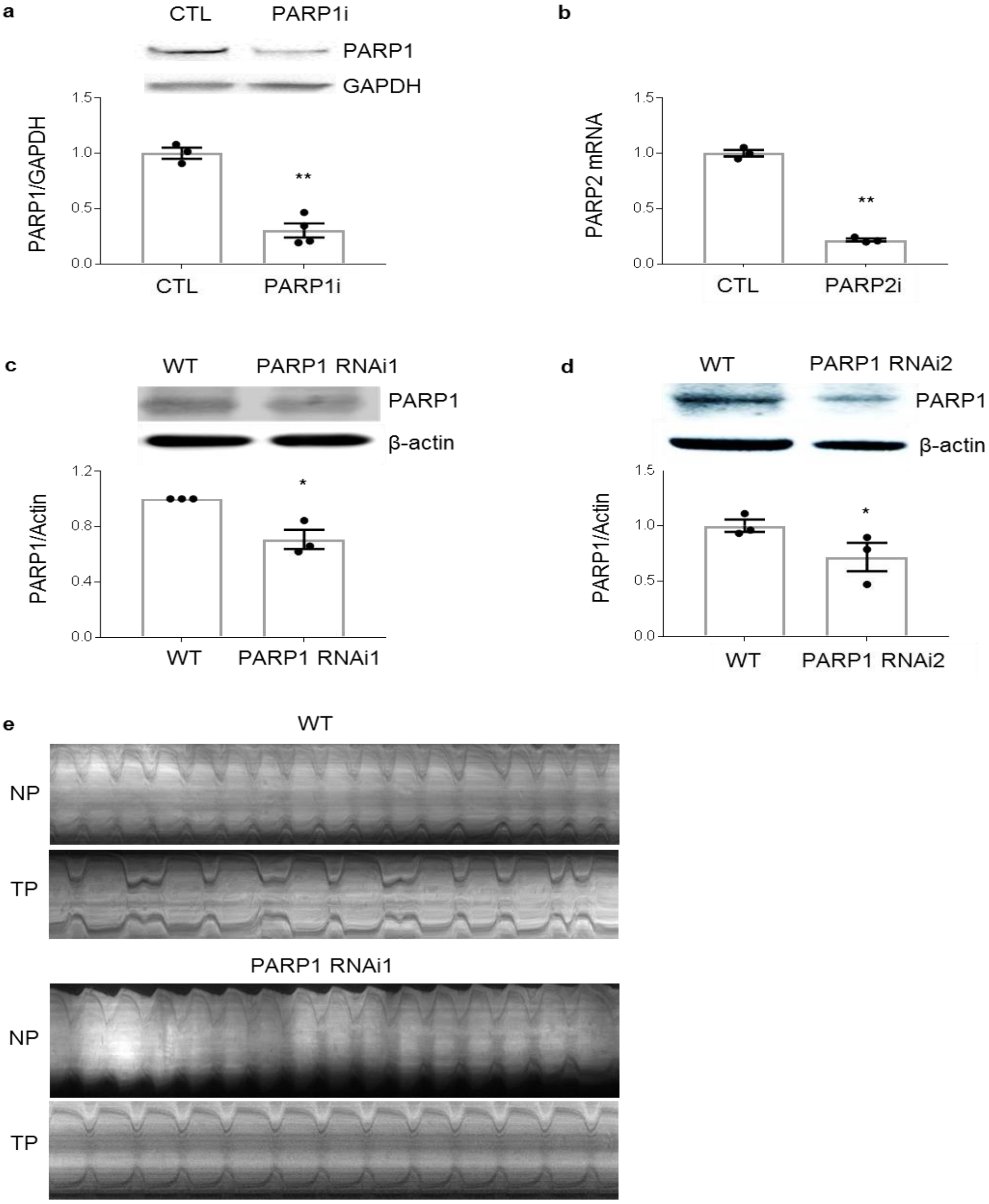

Supplementary Figure 4: PARP1 knockdown in HL-1 cardiomyocytes and *Drosophila* a) Representative Western blot showing significant knockdown of PARP1 in HL-1 cardiomyocytes transfected with PARP1 siRNA (PARP1i) compared to HL-1 cardiomyocytes transfected with scrambled siRNA (CTL). ***P*<0.01 vs CTL. **b**) Quantified reverse-transcription PCR results showing significant knockdown of PARP2 in HL-1 cardiomyocytes transfected with PARP2 siRNA (PARP2i) compared to HL-1 cardiomyocytes transfected with scrambled siRNA (CTL). ***P*<0.01 vs CTL**. c**) Representative Western blot showing significant knockdown of PARP1 in *Drosophila* PARP1i RNA1 (Hand4-GAL4 crossed with UAS-PARP1 shRNA *Drosophila* from VDRC) and **d)** PARP1 RNAi2 (Hand4-GAL4 crossed with UAS-PARP1 shRNA *Drosophila* from BDSC) compared to wild-type (WT: Hand4-GAL4 crossed with wild-type W1118). **P*<0.05 vs WT, N=3 independent experiments. **e**) Representative traces (10 seconds) prepared from high-speed (100 frames per seconds) movies of *Drosophila* prepupae. Movies were made in *Drosophila* prepupae before TP (NP) or after tachypacing (TP). Data are presented as mean ± s.e.m. and two-tailed t-test was used to evaluate differences between groups.

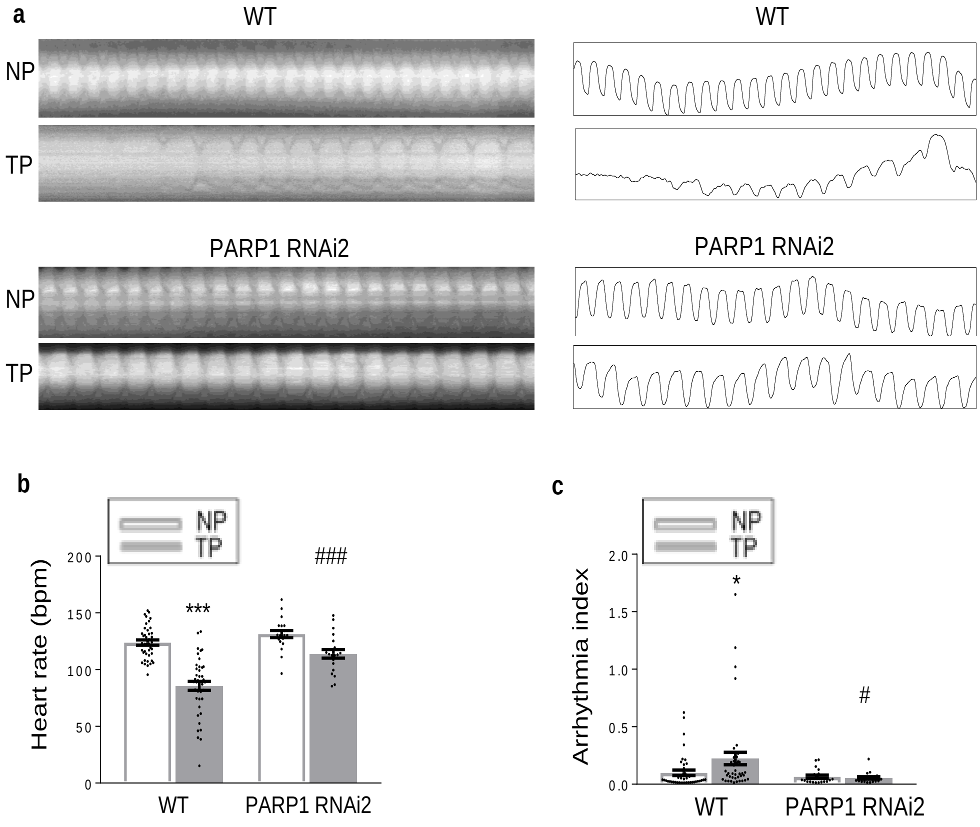

Supplementary Figure 5: PARP1 knockdown protects against tachypacing-induced contractile dysfunction in *Drosophila* a) Representative M-mode cardiography (left) and corresponding heart wall motion traces (right) of 10 s high-speed movies (100 f per s) of *Drosophil*a prepupa. Movies were obtained from non-paced (NP) and tachypaced (TP) *Drosophila* prepupa in wildtype (WT, N=41) and PARP1 knockdown line PARP1 RNAi2 (N=20). **b**) Quantified heart rate (bpm: beats per minute) and **c**) Quantified arrhythmicity index. *P<0.05, ***P<0.001 vs control WT NP, $^{\#}$P<0.05, $^{\#\#\#}$P<0.001 vs WT TP. Data are presented as mean ± s.e.m. and two-tailed t-test was used to evaluate differences between groups.

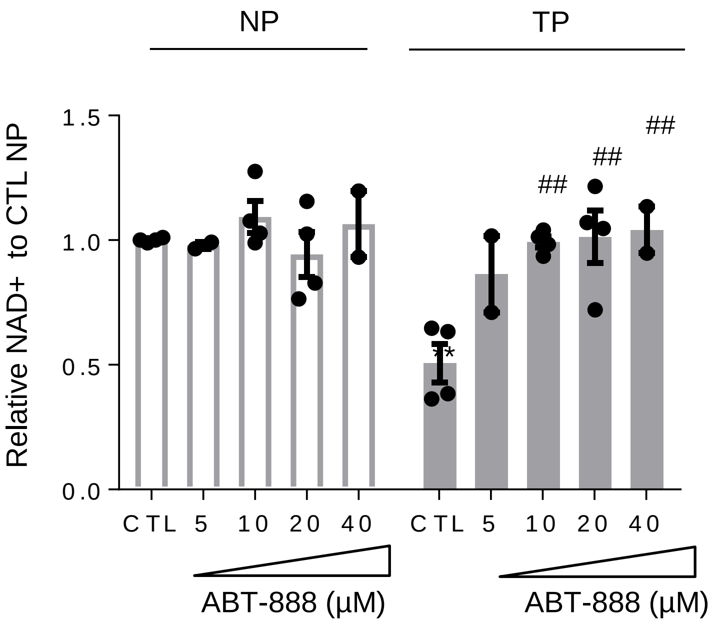

Supplementary Figure 6: The PARP1 inhibitor ABT-888 protects dose-dependently against tachypacing-induced NAD⁺ depletion in HL-1 cardiomyocytes. NAD^+ was measured in control normal paced (NP) and tachypaced (TP) HL-1 cardiomyocytes pretreated with different doses of ABT-888 (5-40 µM) or vehicle (CTL). ABT-888 in, a dose-dependent manner, prevented TP-induced reduction in NAD^+ levels. $^{**}P<0.01$ vs control NP, $^{\#\#}P<0.01$ vs control TP. Data are presented as mean ± s.e.m. and two-tailed t-test was used to evaluate differences between groups.

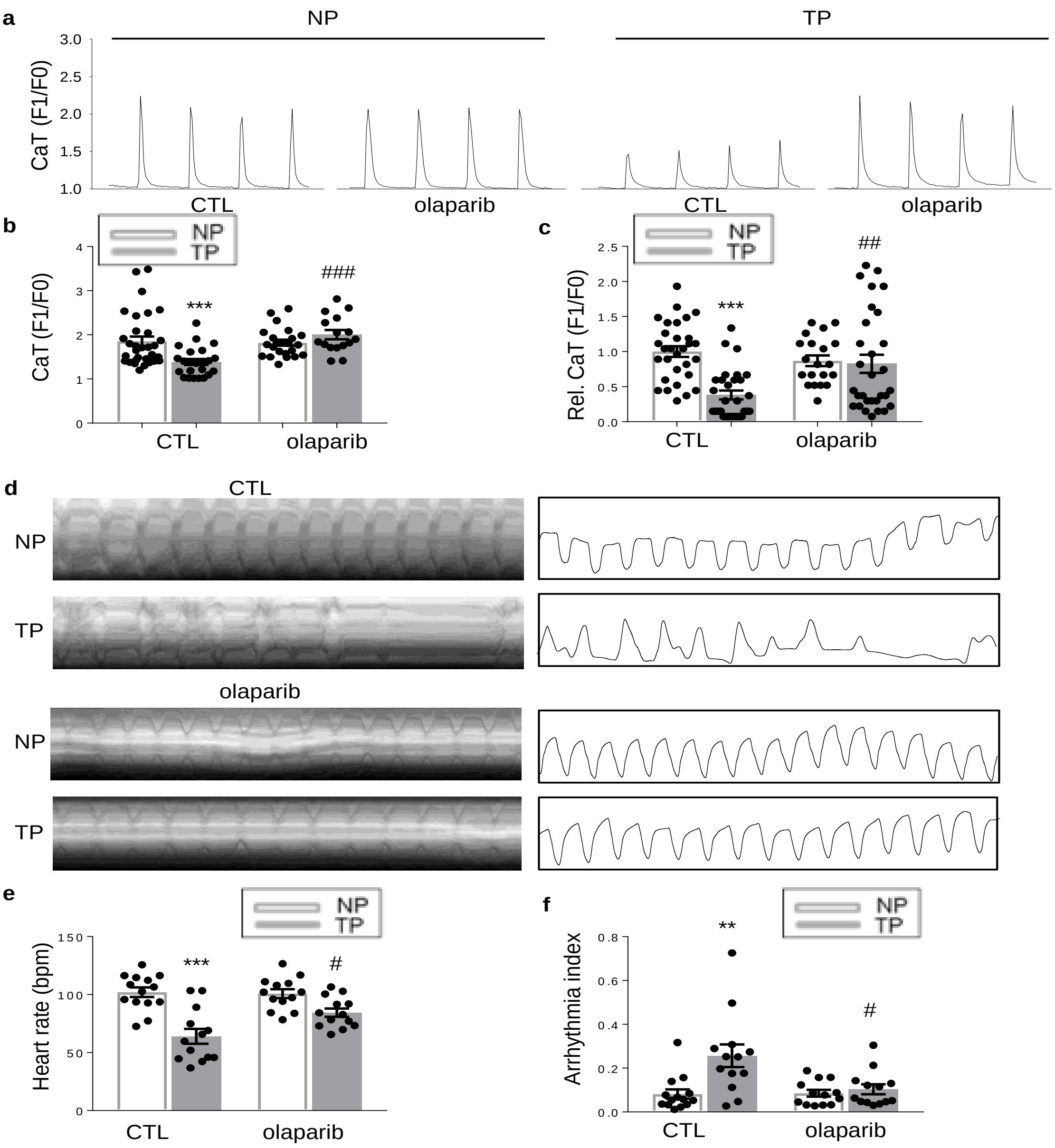

Supplementary Figure 7: PARP1/2 selective inhibitor olaparib prevented tachypacing-induced contractile dysfunction in adult rat atrial cardiomyocytes and *Drosophila*. a) and **b**) Representative CaT traces and quantified CaT amplitude in control non-paced (NP) or tachypaced (TP) rat atrial cardiomyocytes pretreated with olaparib or vehicle DMSO (CTL). ****P*<0.001 vs control NP, ###*P*<0.001 vs control TP, N=32 atrial cardiomyocytes for NP CTL, N=23 for NP olaparib, N=23 for CTL TP, N=16 for TP olaprib. **c**) Relative CaT amplitude in NP or TP HL-1 cardiomyocytes pretreated with olaparib or vehicle DMSO (CTL). ****P*<0.001 vs control NP, ##*P*<0.01 vs control TP, N=30 atrial cardiomyocytes for CTL NP, CTL TP and olaparib TP, N=20 for olaparib TP. **d**) M-mode traces (left) and heart wall motion traces (right) of 10 s prepared from high-speed movies of *Drosophila* prepupae. *Drosophilas* were pretreated with olaparib or vehicle DMSO (CTL). Movies were made from non-paced (NP) and tachypaced (TP) *Drosophila* prepupae. **e**-**f**) Quantified heart rate (bpm: beats per minute), arrhythmicity index. ***P*<0.01, ****P*<0.001 vs control BP, #*P*<0.05 vs control TP, N=14 *Drosophila* prepupae for CTL, N=13 for olaparib. Data are presented as mean ± s.e.m. and two-tailed t-test was used to evaluate differences between groups.

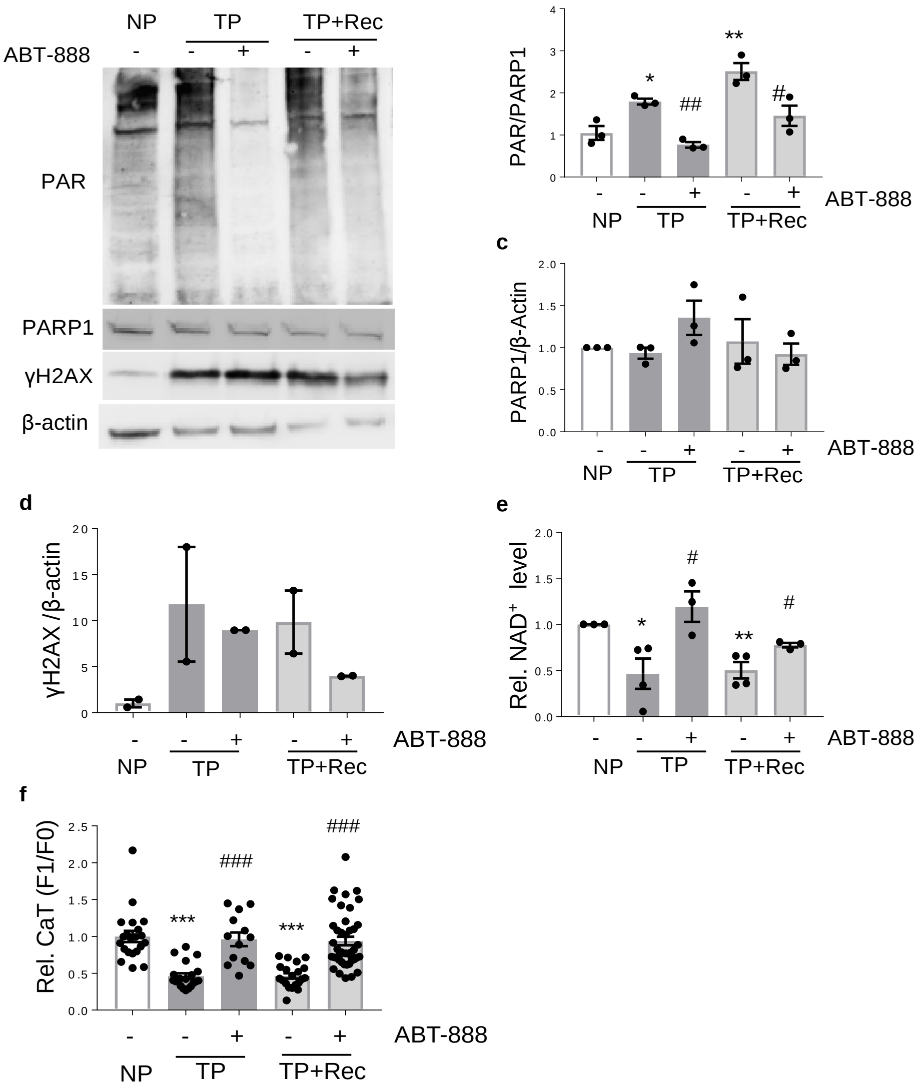

Supplementary Figure 8: PARP1 inhibitor prevents and reverses tachypacing-induced contractile dysfunction in HL-1 cardiomyocytes a) Representative Western blot showing PAR, PARP1 protein, and γH2AX levels in control non-paced (NP), and tachypaced (TP) group with or without ABT-888 pretreatment 12 h before tachypacing, and in TP plus 24 h recovery group (TP+Rec) with or without ABT-888 posttreatment. **b**) Quantified data showing significant increase in PAR in TP and TP+Rec groups, which was inhibited by ABT-888. N=3 independent experiments. **c**) Quantified data showing comparable PARP1 protein levels for all conditions as indicated. N=3 independent experiments. **d**) Quantified data showing increased γH2AX levels, indicating induction of DNA damage in TP and TP+Rec groups with or without ABT-888 treatment. N=2 independent experiments. **e**) Quantified data showing significant decreased NAD^+ levels in TP and TP+Rec groups, which are inhibited or recovered, respectively, by ABT-888 treatment. N=4 independent experiments. **f**) Quantified data showing significant reduction of CaT in TP and TP+Rec groups, which were prevented or reversed by ABT-888 treatment, respectively. N=21 cardiomyocytes for NP, N=20 for TP, N=13 for TP+ABT-888, N=20 for TP+Rec, N=42 for TP+Rec+ABT-888. **P*<0.05, ***P*<0.01, ****P*<0.001, vs NP, $^{\#}P<0.05$, $^{\#\#}P<0.01$, $^{\#\#\#}P<0.001$ TP/TP+Rec without ABT treatment vs TP/TP+Rec with ABT-888 treatment, respectively. Data are presented as mean ± s.e.m. and two-tailed t-test was used to evaluate differences between groups.

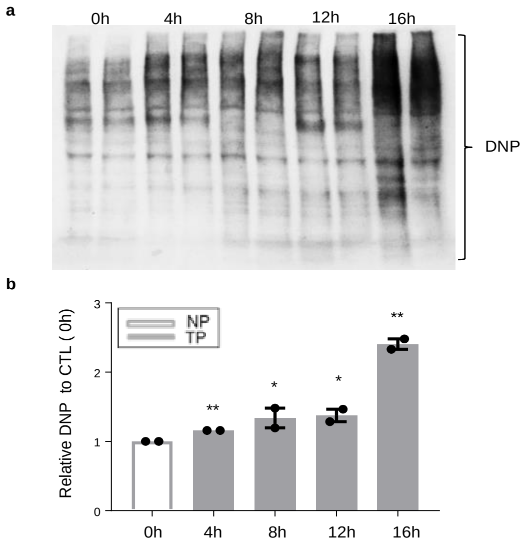

Supplementary Figure 9: Tachypacing induces oxidative stress in HL-1 cardiomyocytes. **a**) Representative Western blot of DNP and **b**) quantified data of relative DNP levels (representing oxidized protein) in HL-1 cardiomyocytes at different duration of tachypacing (TP) showing significant induction of oxidation of proteins from 4 h TP. *P<0.05, **P<0.01 vs control 0 h. Data are presented as mean ± s.e.m. and two-tailed t-test was used to evaluate differences between groups.

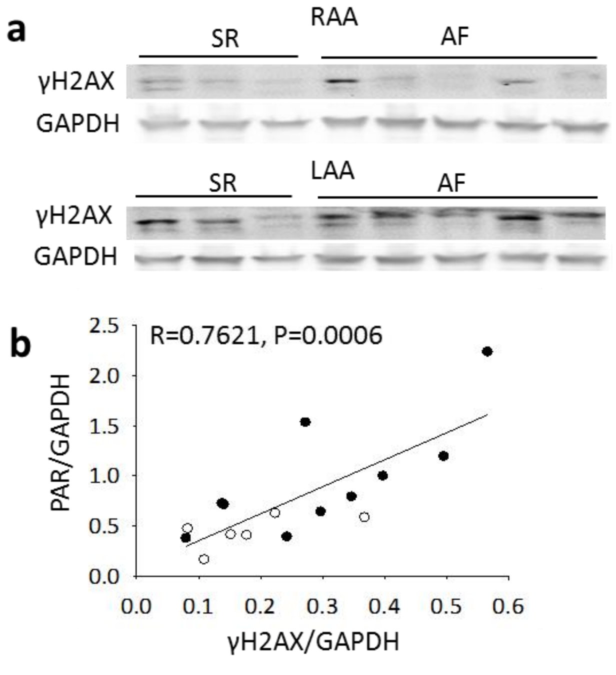

Supplementary Figure 10: γH2AX and PAR levels correlate significantly in AF patients. **a**) Representative Western blot of γH2AX in RAA and LAA tissue of SR and AF patients. **b**) Correlation of PAR and γH2AX levels, showing significant correlation between PARP1 activity (PAR levels) and DNA damage (γH2AX levels). SR: open circle and AF: filled circle. N=3 for SR RAA, N=3 for SR LAA, N=5 for AF RAA and N=5 for AF LAA.

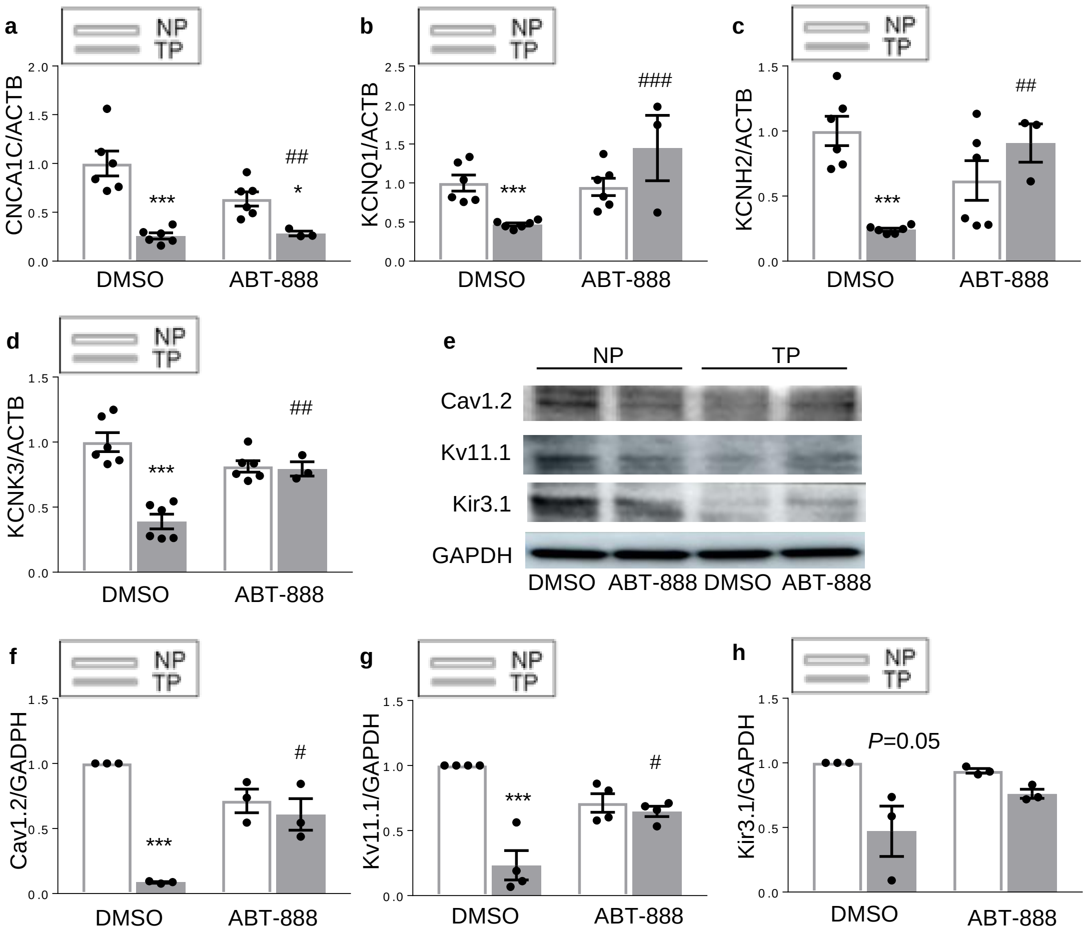

Supplementary Figure 11: PARP1 inhibitor prevents tachypacing-induced ion channel remodeling. **a-e**) Quantified PCR analysis showing that the transcripts of α-subunit of L-type Ca^{2+} (a) and three K^+ channels (b-d) are all significantly reduced by tachypacing (TP). PARP1 inhibitor ABT-888 significantly prevented all TP-induced reductions. ****P*<0.001 vs DMSO NP, $^{##}$*P*<0.01, $^{###}$*P*<0.001 vs DMSO TP, N=6 experiments for DMSO NP, ABT-888 NP and DMSO TP, N=3 experiments for ABT-888 TP for all the channels. **e-h**) Representative Western blot image and quantified data confirming the findings from qPCR. The α-subunit of L-type Ca^{2+} (Cav1.2) and K^+ channel protein levels (Kv11.1 and Kir3.1) were significantly reduced by TP, which were prevented by ABT-888 treatment. **P*=0.05 vs DMSO NP ****P*<0.001 vs DMSO NP, $^{#}$*P*<0.05 vs DMSO TP, N=3 experiments each group (DMSO NP/TP, ABT-888 NP/TP) for Cav1.2 and Kir3.1, N=4 experiments each group for Kv11.1. Data are presented as mean ± s.e.m. and two-sided t-test was used to evaluate differences between groups.

Supplementary Figure 12: Full, un-cropped Western blots as presented in main article Figures and Supplementary Figures. Boxes indicate cropped regions.

Fig. 1a and Supplementary Figure 1a

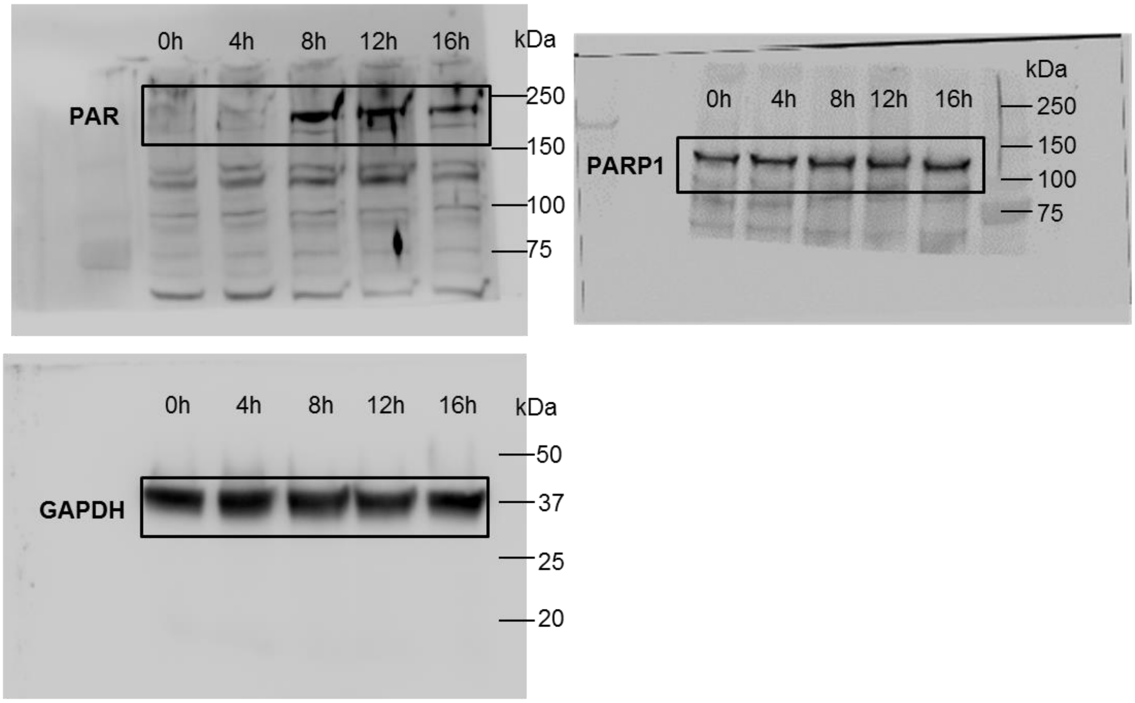

Fig. 1g

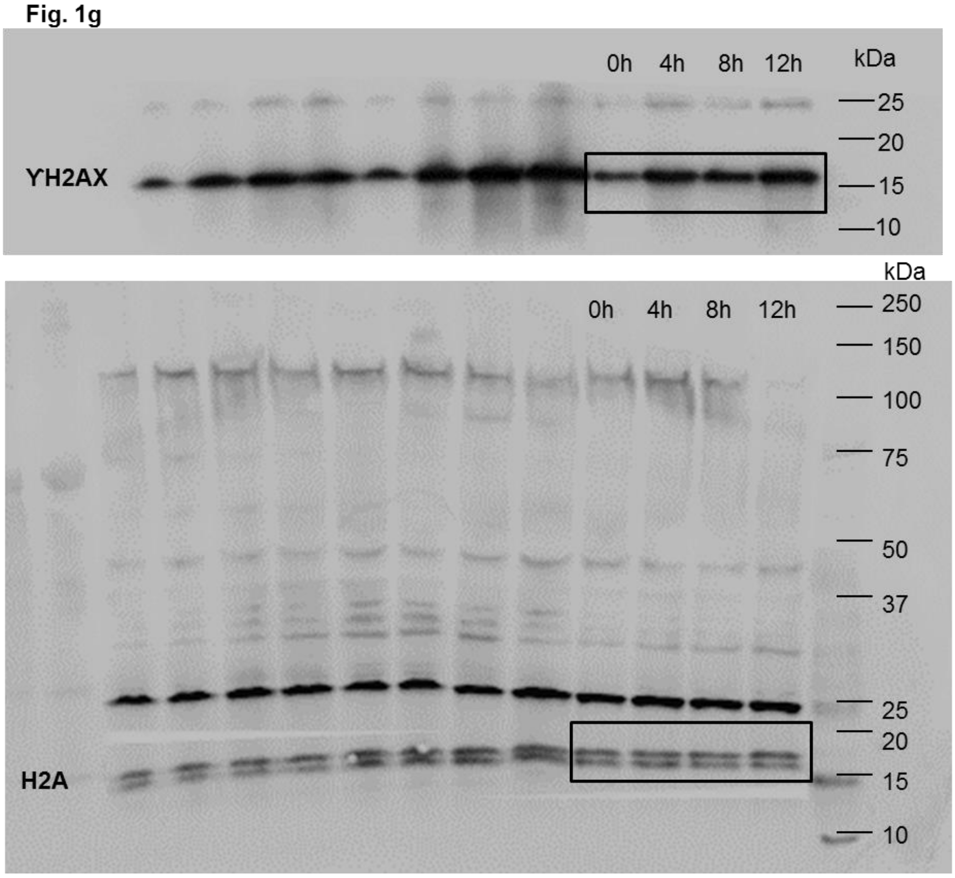

Fig. 4a

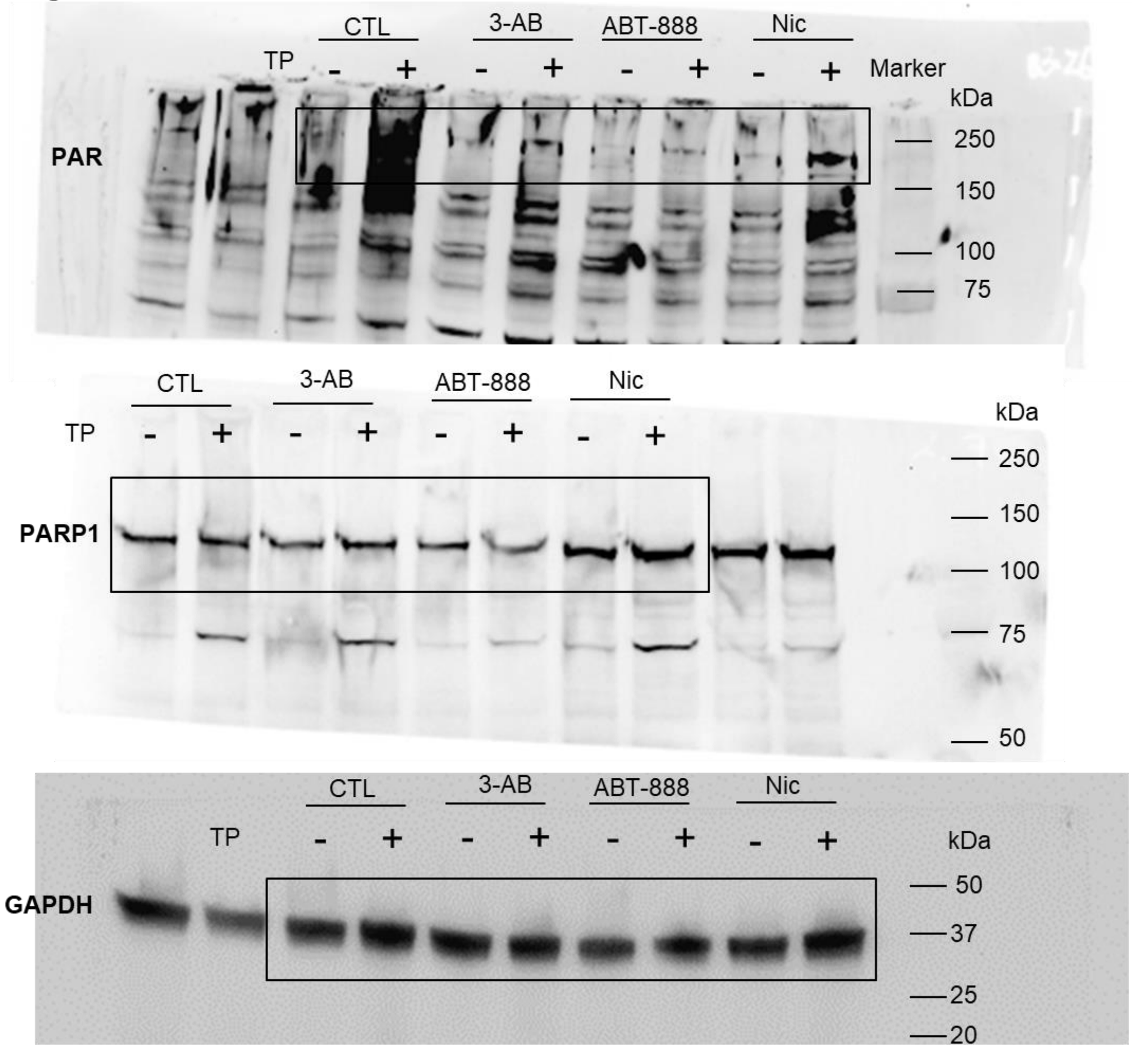
CTL
3-AB
ABT-888
Nic
TP
- + - + - + - +
Marker
kDa
PAR
250
150
100
75
PARP1
50
GAPDH
37
25
20

Fig. 6a

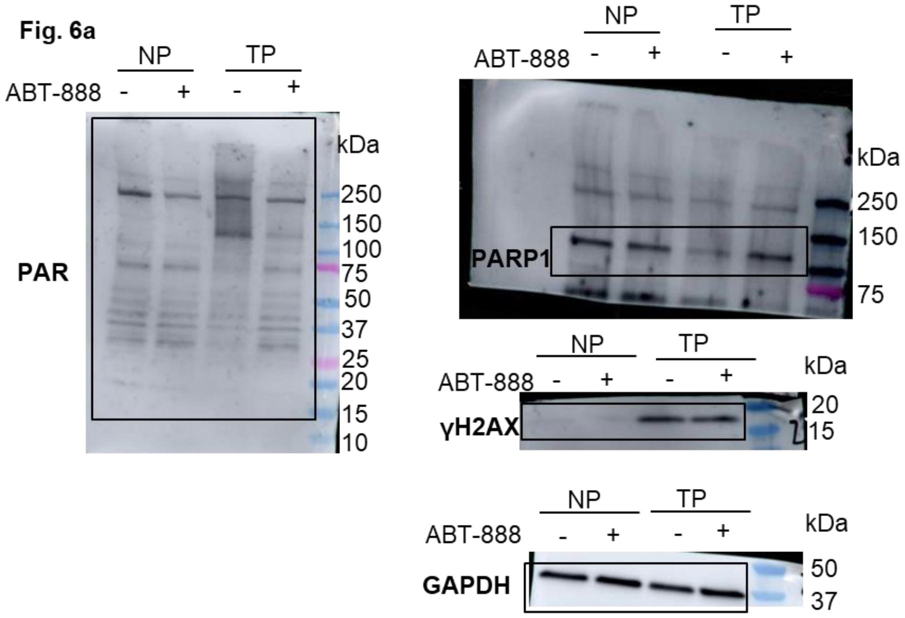
NP
TP
ABT-888
- + - +
PAR
kDa
250
150
100
75
50
37
25
20
15
10
PARP1
γH2AX
GAPDH

Fig. 7a

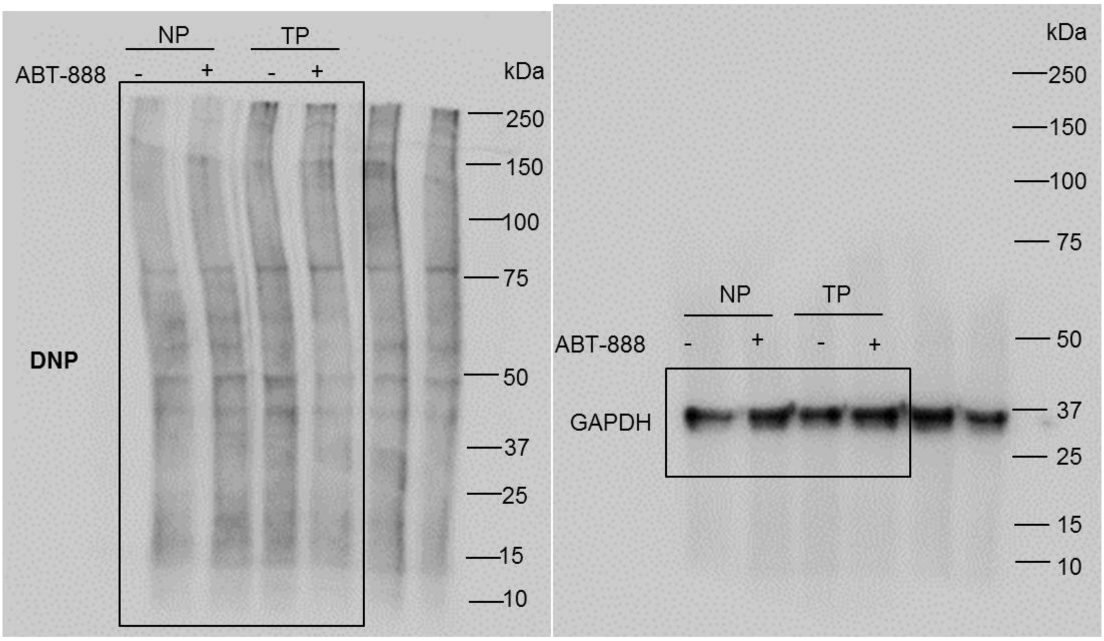
NP
TP
ABT-888
- + - +
kDa
250
150
100
75
50
37
25
15
10
DNP
GAPDH

Fig. 8a

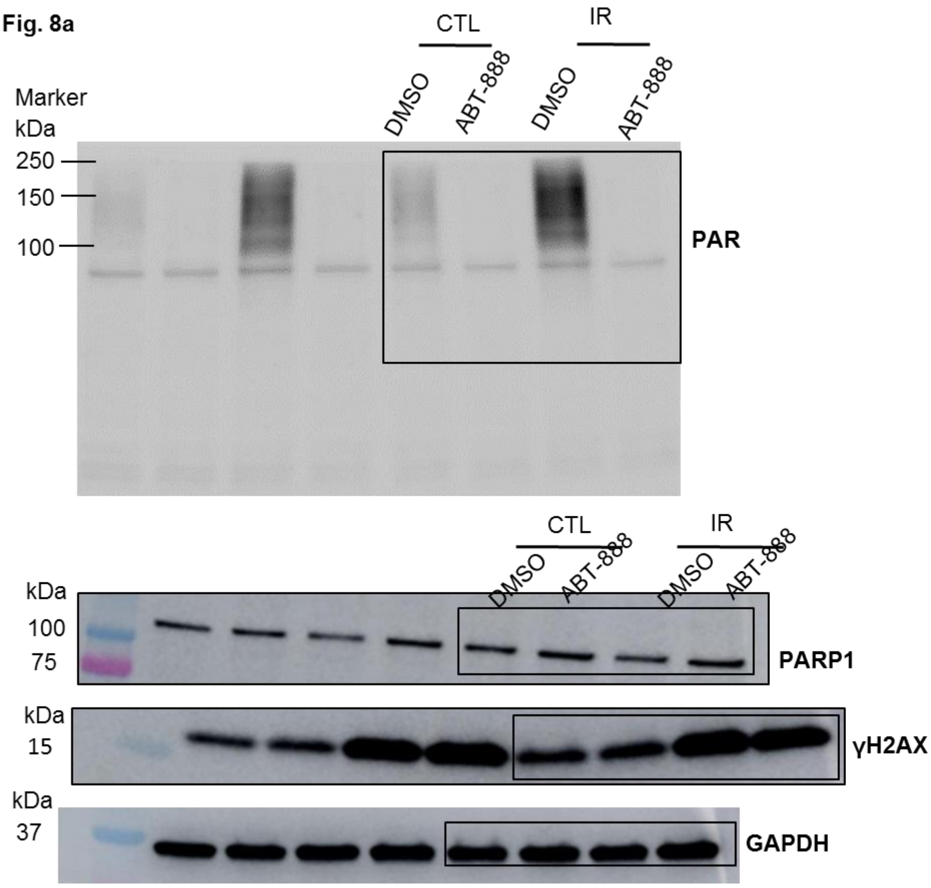
CTL
IR
DMSO
ABT-888
DMSO
ABT-888
Marker
kDa
250
150
100
PAR
kDa
100
75
PARP1
kDa
15
γH2AX
kDa
37
GAPDH

Fig. 9a

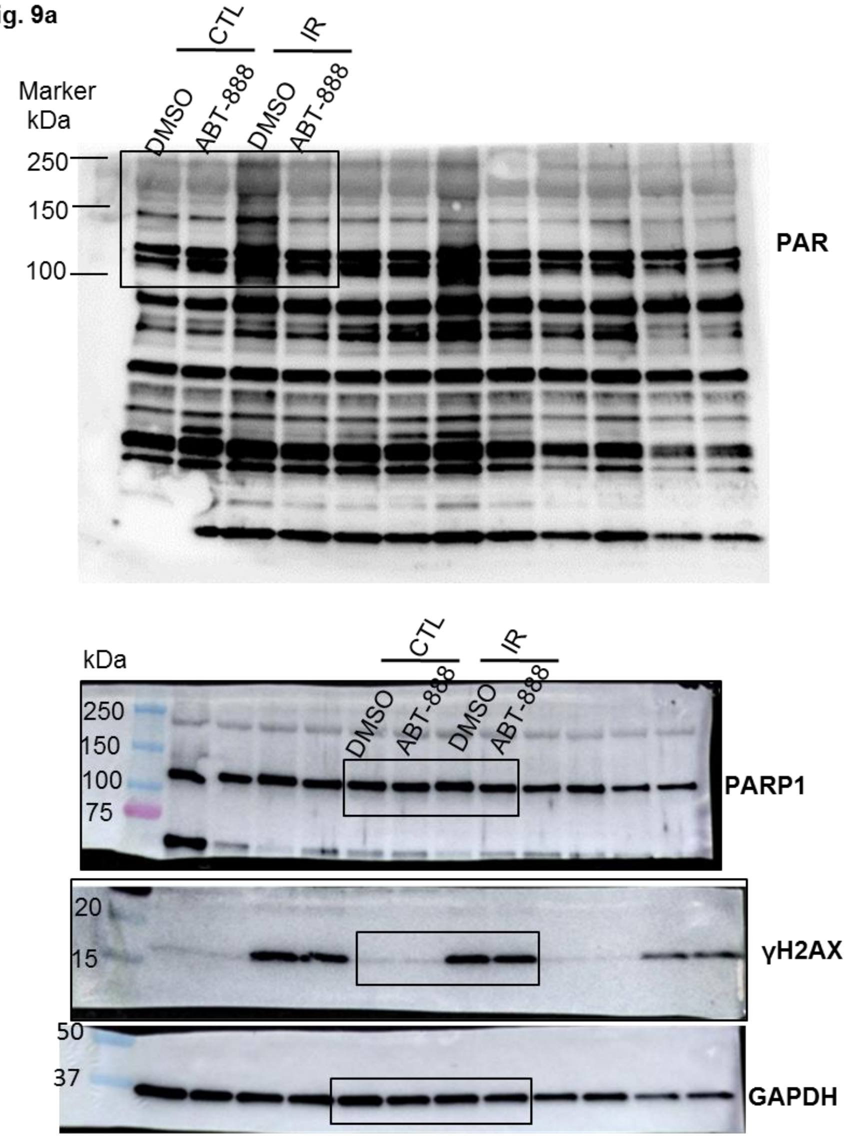
CTL
IR
Marker
kDa
DMSO
ABT-888
DMSO
ABT-888
250
150
100
PAR
kDa
CTL
IR
DMSO
ABT-888
DMSO
ABT-888
250
150
100
75
PARP1
20
15
γH2AX
50
37
GAPDH

Supplemental Figure 2 a and f

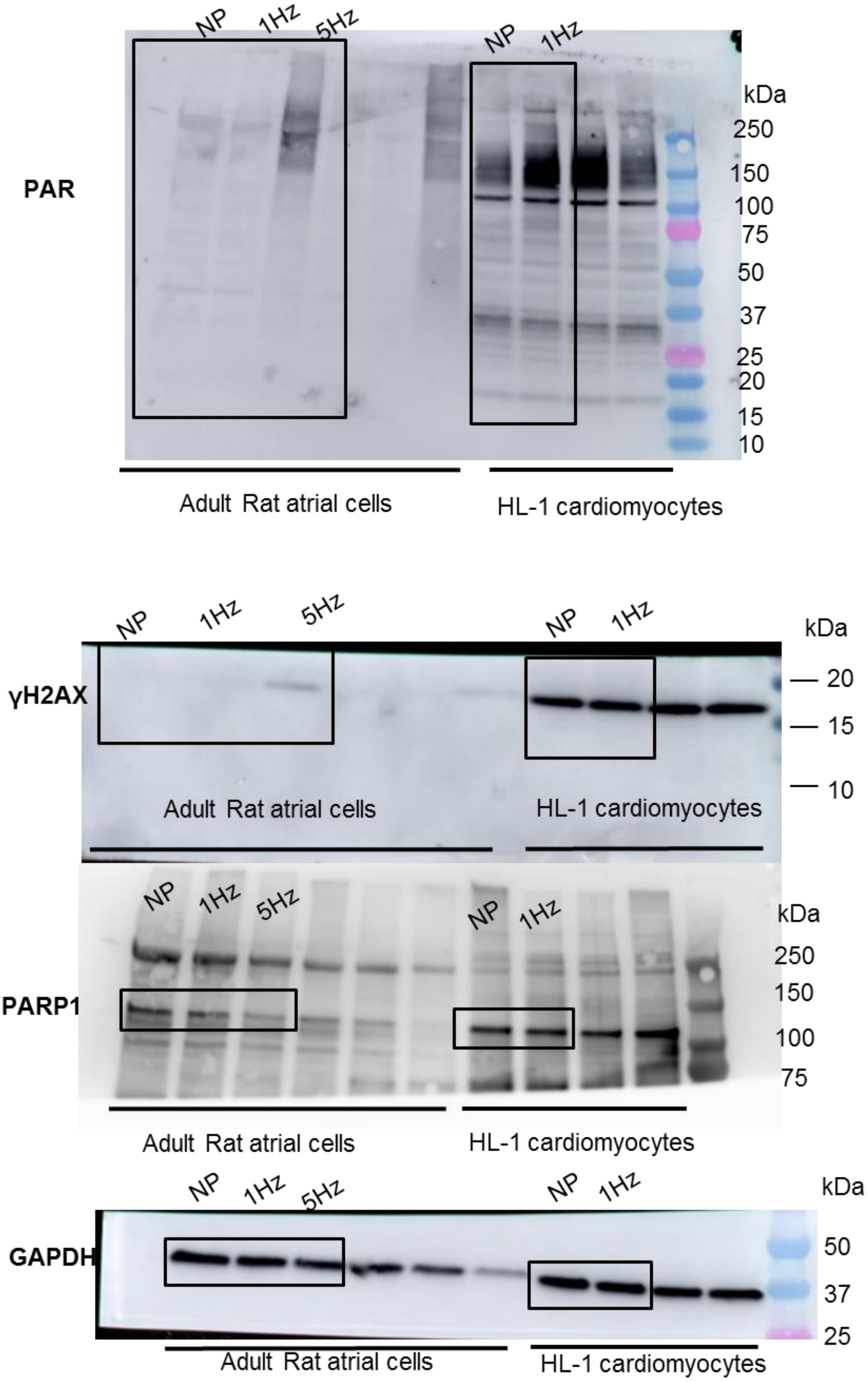

Fig. 10a

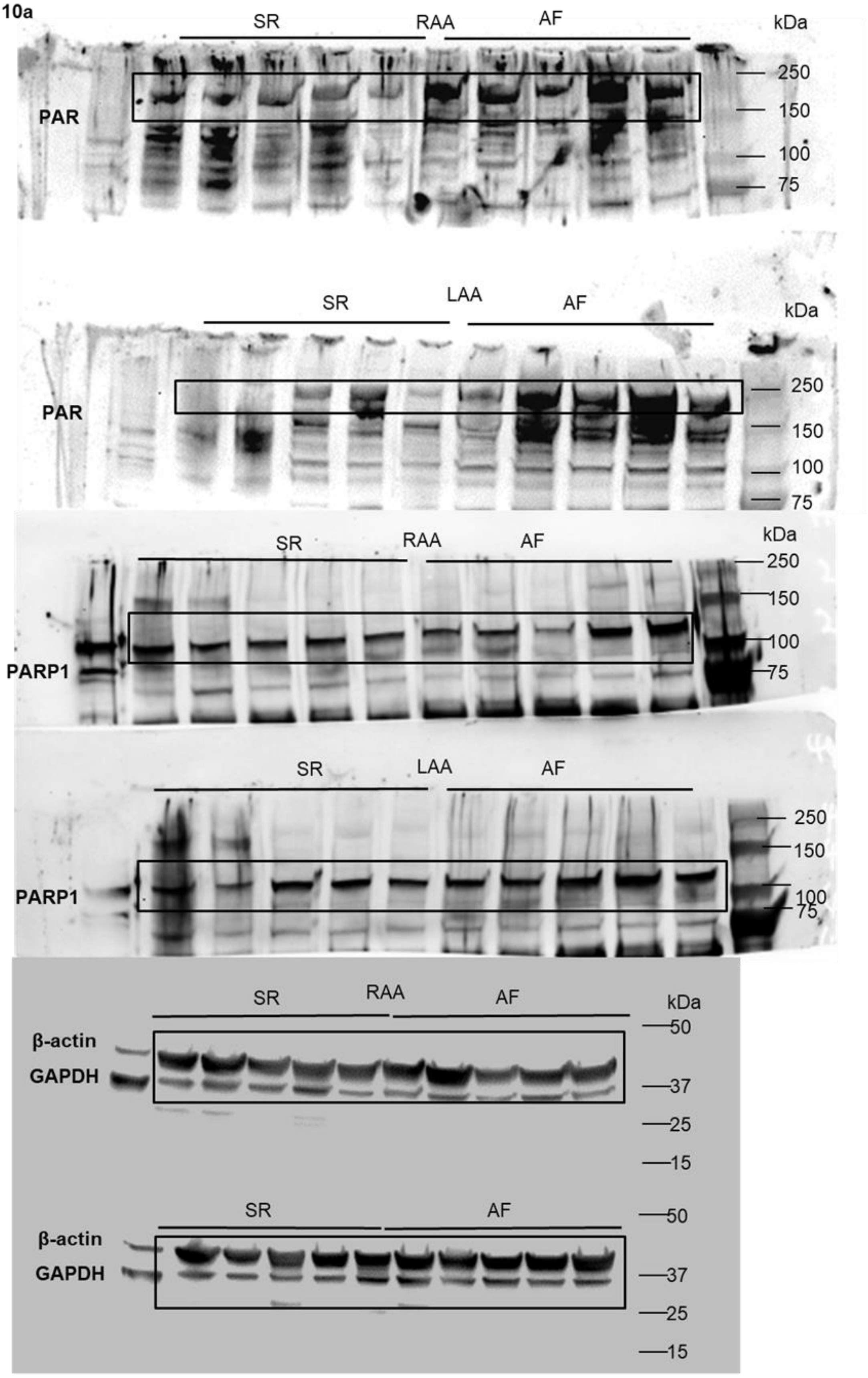
SR
RAA
AF
kDa
PAR
250
150
100
75
SR
LAA
AF
kDa
PAR
250
150
100
75
SR
RAA
AF
kDa
PARP1
250
150
100
75
SR
LAA
AF
PARP1
250
150
100
75
SR
RAA
AF
kDa
β-actin
GAPDH
50
37
25
15
SR
AF
β-actin
GAPDH
50
37
25
15

Supplemental Figure 4c

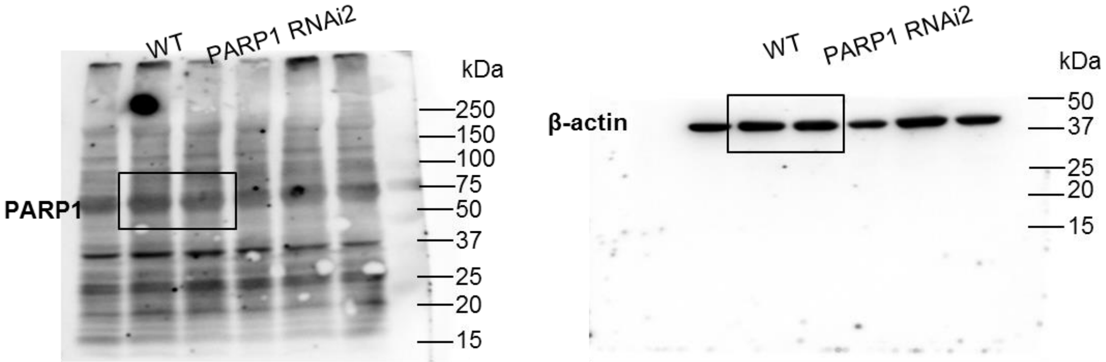

Supplemental Figure 4a

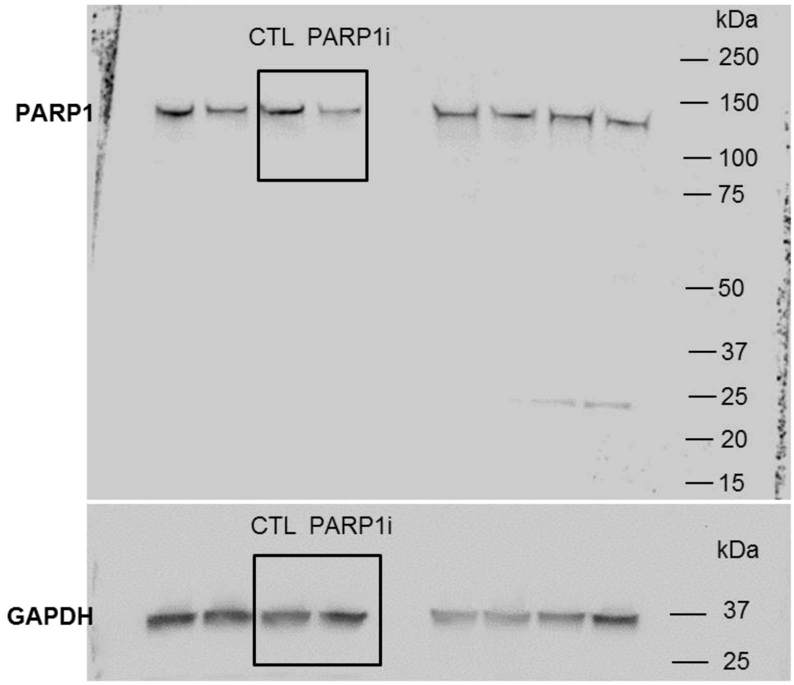

Supplemental Figure 4d

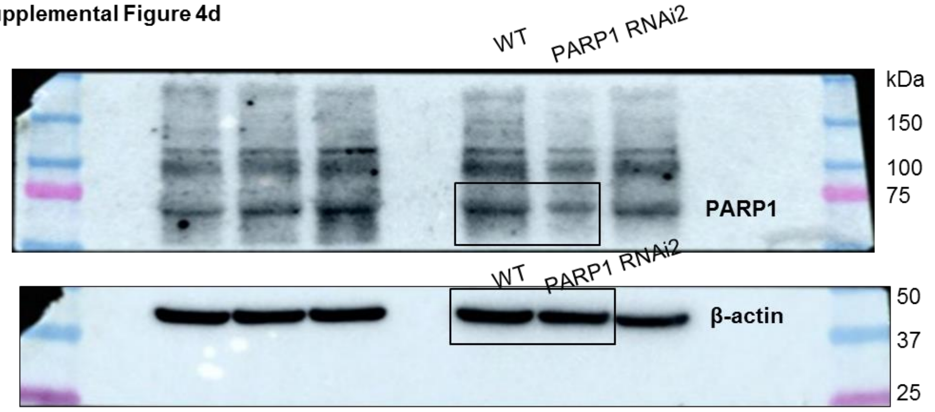

Supplemental Figure 8a

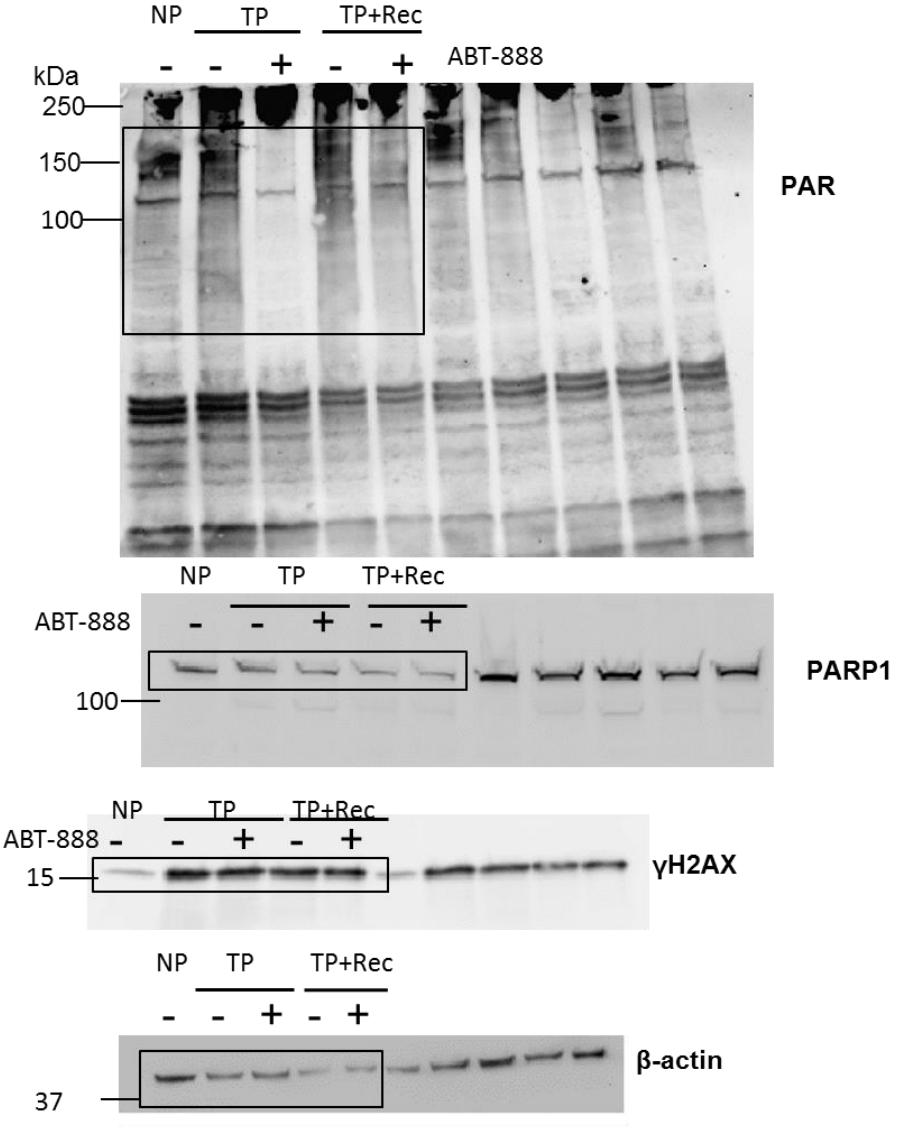

Supplemental Figure 9a

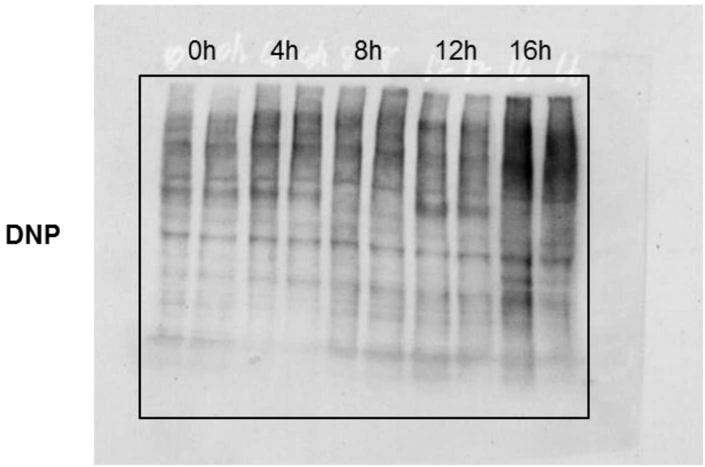

Supplemental Figure 10a

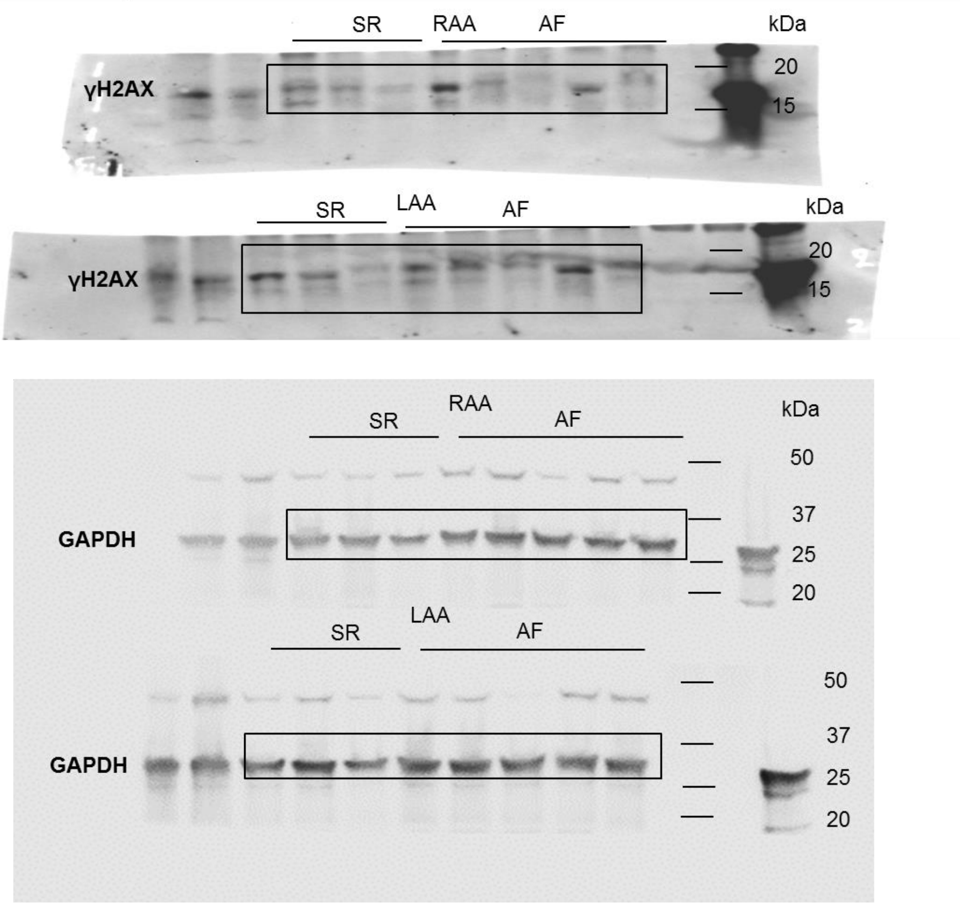

Supplemental Figure 11a

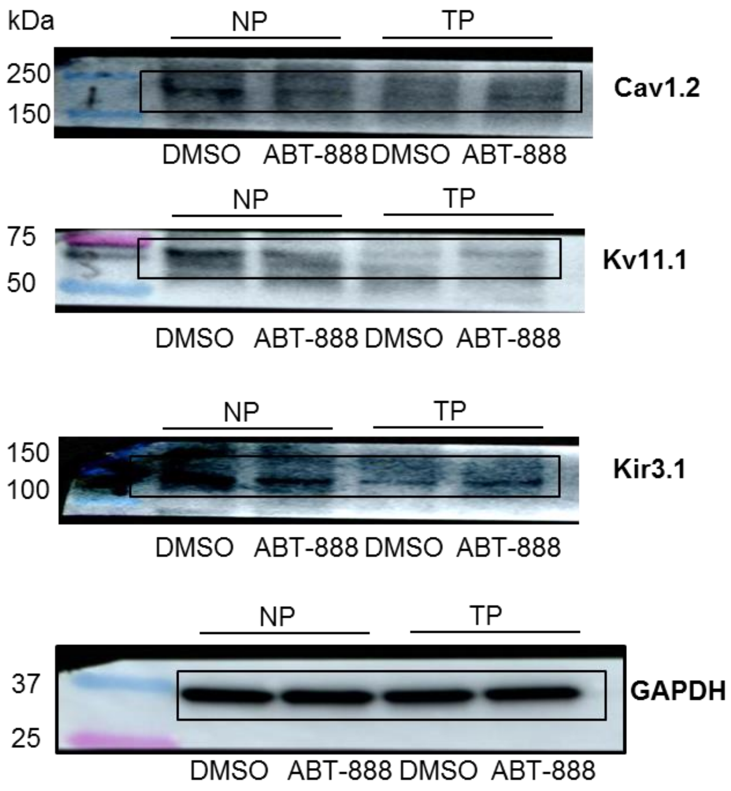

Supplement: Supplementary file 1 — Supplementary Information [file 41467_2019_9014_MOESM1_ESM.pdf]
